# Supplementary material for: Characterization and quantitative trait locus mapping of late-flowering from a Thai soybean cultivar introduced into a photoperiod-insensitive genetic background
Source: PLoS One. 2019 Dec 5;14(12):e0226116. doi: 10.1371/journal.pone.0226116 (PMC6894811; doi:10.1371/journal.pone.0226116)
Supplement: Attachment — Submitted filename: Responses to the reviewers comments final.docx [file pone.0226116.s004.docx]

We appreciate the reviewer’s comments and suggestions. We revised the points indicated as follows:

1. Please provide genomic positions of gene specific markers in table 1?
(Response) We added the genomic positions of SNPs targeted by gene-specific markers to Table 1, and added the sentence ‘Genomic positions for gene-specific markers are presented within parentheses’ as a footnote.

2. No information provided on the genes predicted between left marker and right marker confidence intervals of identified QTLs. Are there only single gene present in C.I. of each of identified QTL?
(Response) We have provided information on the annotated genes in the intervals between the left and right markers in a new supplemental table, ‘S2 Table’ (thereby, the original S2 Table was changed to S3 Table in the revised manuscript), and described the numbers of annotated genes in the intervals in the ‘Segregation of flowering time and QTL analysis’ subsection of the Results section in the revised manuscript, as follows:

“The intervals between the left and right markers contained 30, 50 and 167 annotated genes for qDTF-16-1, qDTF-16-2 and qDTF-10, respectively, in the reference genome Williams 82.v2 (S2 Table).”

In addition, we corrected the original sentence “The co-localization with gene-specific DNA markers suggested that *qDTF-10*, *qDTF-16-1* and *qDTF-16-2* most likely corresponded to *E2*, *FT5a* and *FT2a*, respectively (Table 1).” in the ‘QTLs for the LF habit in the photoperiod-insensitive genetic background’ subsection of the Discussion section as follows:

“*qDTF-10*, *qDTF-16-1* and *qDTF-16-2* were co-localized with gene-specific DNA markers for *E2*, *FT5a* and *FT2a*, respectively (Table 1). The intervals between the left and right markers of respective QTLs contained from 30 annotated genes in *qDTF-16-1* to 167 annotated genes in *qDTF-10* annotated genes including several orthologues of *Arabidopsis* flowering genes (S2 Table). However, these three genes may be probable candidates responsible for the QTLs, because their allelic effects on flowering have been revealed in diverse genetic backgrounds [8, 9, 20, 22, 26, 28, 67].” Here we added a new reference as reference number 67 (Wang Y, Gu Y, Gao H, Qiu L, Chang R, Chen S, et al. Molecular and geographic evolutionary support for the essential role of GIGANTEAa in soybean domestication of flowering time. BMC Evol Biol. 2016; 16: 79). We added this citation to the list of the references.

3. Please correct ‘IcMAPPING’ to ‘IciMapping’ in Line no. 321 of Page no. 14
(Response) We corrected our mistake as indicated by the reviewer.

4. Transcript profiles of FT2a and FT5a were analyzed but why not E2? Although E2 was not identified in all four environments, transcript profiles in SD and LD environments may have supported it.

(Response) Thank you for your comment. We added the results of the E2 expression analysis to the revised manuscript. Accordingly we added information on E2 to the ‘Expression analysis’ subsection of the Materials and Methods section and to the ‘Transcript profiles for FT2a, FT5a and E2’ subsection of the Results section. The primers were also added to S1 table. We made a new figure (Figure 8) based on the results of the expression analysis, and described the results in the ‘Transcript profiles for FT2a, FT5a and E2’ subsection of the Results section in the revised manuscript, as follows:

“We also examined the transcript profile of *E2* as a possible candidate for *qDTF-10* (Fig 8A–B). The transcript abundance of *E2* peaked at 12 ZT and was repressed at 3 and 21 ZT under all conditions. AK16, which possessed the functional *E2* allele, exhibited higher expression levels than AGS292, which possessed the dysfunctional *e2* allele, under all conditions, except for the R-LD at the second trifoliate-leaf stage. The *E2* expression was upregulated at both growing stages in R-LD and at the fourth trifoliate-leaf stage under 32°C conditions, compared with SD and the other two thermal conditions (18°C and 25°C), respectively. However, there was no marked difference in the responses to different photoperiods and thermal conditions between AGS292 and AK16.”

In addition to the corrections in response to the reviewer’s comments, we made five corrections in the revised manuscript, as follows:

1. [TITLE] We would like to change the title of the paper from the original ‘Characterization and quantitative trait locus mapping of late-flowering in a Thai soybean cultivar with a photoperiod-insensitive genetic background’ to ‘Characterization and quantitative trait locus mapping of late-flowering from a Thai soybean cultivar introduced into a photoperiod insensitive genetic background’ in the revised one (the corrected portions were underlined). We believe the original title may lead the reader to believe that the Thai cultivar itself possesses a photoperiod-insensitive genetic background.

2. [Discussion, lines 515 to 518] We mad changes to the following original sentences: “The intervals with the largest LOD scores included the gene-specific marker for FT5a under all the conditions, except R-LD conditions, where the greatest LOD score was located in the interval of the next two SNPs, SNP 4900158 and SNP….”. We corrected the term “R-LD” to “FR-LD” and added the appropriate identifying number after SNP, as follows (the corrected portions are underlined): “The interval with the largest LOD scores varied with the environments tested. The intervals with the largest LOD scores included the gene-specific marker for FT5a under all the conditions, except FR-LD conditions, where the greatest LOD score was located in the interval of the next two SNPs, SNP 4900158 and SNP 53735087…”.

3. [Discussion, line 547] We added the citations ([this study, 9]) after “nonsense mutations”, as follows (the corrected portions are underlined): “The LJ in soybean might, therefore, reflect a slower floral evocation process owing to the depressed function of FT2a resulting from missense or nonsense mutations [this study, 9] or from …”.

4. [Figure 4] We corrected the order of SNP markers in Chromosome 10, where the QTL for flowering (corresponding to E2) was located, because the SNPs in this chromosome in the original Figure 4 was ordered in the opposite direction of their genomic positions in the Williams 82 reference genome. In the revised manuscript, we presented the corrected figure with the SNP markers in the correct order relative to the reference genome.

5. [Table 1] We corrected the QTL name from qFT to qDTF. In addition, the SNPs for the left and right markers at qDTF-10 in ND and FR-LD were placed in the correct order relative to their genomic positions in the Williams 82 reference genome.

We also corrected wrong citations as follows:

1. Line 69 (Introduction); We corrected 9 to 10.

2. Line 449 (Discussion); We corrected 61–63 to 63–65.

3. Line 452 (Discussion); We corrected 54 to 66. Reference 54 was the incorrect citation. The proper reference is ‘Cober ER, Stewart DW, Voldeng HD. Photoperiod and temperature responses in early-maturing near-isogenic soybean lines. Crop Sci. 2001; 41: 721–727’. We added this citation to the list of the references. The original reference numbers of 66-73 were thus changed to the reference number of 68-75 in the revised manuscript, by adding two references (66 and 67).

4. Line 484 (Discussion); We corrected 12 to 13.
